# Antimicrobial, Antioxidant, and Wound Healing Properties of *Kigelia africana* (Lam.) Beneth. and *Strophanthus hispidus* DC.

**DOI:** 10.1155/2013/692613

**Published:** 2013-04-11

**Authors:** Christian Agyare, Anita Serwaa Dwobeng, Nicholas Agyepong, Yaw Duah Boakye, Kwesi Boadu Mensah, Patrick George Ayande, Martin Adarkwa-Yiadom

**Affiliations:** ^1^Department of Pharmaceutics, Faculty of Pharmacy and Pharmaceutical Sciences, Kwame Nkrumah University of Science and Technology, Kumasi, Ghana; ^2^Department of Pharmacology, Faculty of Pharmacy and Pharmaceutical Sciences, Kwame Nkrumah University of Science and Technology, Kumasi, Ghana; ^3^Department of Human Biology and Nursing, School of Biological Sciences, University of Cape Coast, Cape Coast, Ghana; ^4^Drugs and Forensic Laboratory, Ghana Standards Authority, Accra, Ghana

## Abstract

Microbial infections of various types of wounds are a challenge to the treatment of wounds and wound healing. The study was to investigate antimicrobial and antioxidant properties of methanol leaf and stem bark extracts of *Kigelia africana* and methanol leaf and root extracts of *Strophanthus hispidus* and also to determine wound healing properties of the extracts. The antimicrobial activities of the methanol extracts were determined against two Gram-positive and two Gram-negative bacteria and a fungus using agar diffusion and micro-dilution methods. The antioxidant activity was determined using 1,1-diphenyl-2-picryl–hydrazyl (DPPH) method. The influence of the extracts on rate of wound closure was investigated using the excision wound model and histopathological investigation of treated and untreated wound tissues performed. The MICs of leaf extract of *K. africana* against test organisms were 2.5–7.5 mg/mL and stem bark extract were 2.25–7.5 mg/mL. The leaf extract of *S. hispidus* had MIC range of 2.5–7.5 mg/mL and 2.5–10 mg/mL for root extract. The IC_50_ of leaf and stem bark extracts of *K. africana* were 56.9 and 13.7 **μ**g/mL, respectively and leaf and root of *S. hispidus* were 49.8 and 45.1 **μ**g/mL, respectively. *K. africana* extracts (7.5% w/w) showed significant (*P* < 0.05) wound contraction at day 7 with 72% of wound closure whiles significant (*P* < 0.05) wound contractions were observed on day 11 for stem bark of *K. africana*, leaf and root extracts of *S. hispidus*. Wound tissues treated with the extracts showed improved collagenation, re-epitheliazition and rapid granulation formation compared with untreated wound tissues. The extracts were found to contain alkaloids, saponins, tannins, flavonoids, carbohydrates, and sapogenetic glycosides. The HPLC finger-printing of the extracts were developed. The leaf, stem bark and root extracts of *K. africana* and *S. hispidus* exhibited antimicrobial, antioxidant, and enhanced wound healing properties and these may justify the medicinal uses of the plants for treatment of microbial infections and wounds.

## 1. Introduction

Wound is most commonly used when referring to injury to the skin or underlying tissues or organs by a blow, cut, missile, or stab. Wound also includes injury to the skin caused by chemicals, cold, friction, heat, pressure and rays, and manifestation in the skin of internal conditions, for example, pressure sores and ulcers [1]. Wounds have a tremendous impact on the healing healthcare economy. Chronic wounds represent a major health burden and drain on the healthcare resources in the world including Ghana [2].

A major problem with wounds is the high risk of infection; hence, if an agent active against these microorganisms causing the infection is used in the healing process, it will then help to reduce the risk of infection and the overall time for wound healing can be reduced significantly. For example, it is very easy for bacteria to enter through the broken skin and penetrate the rest of the body. Bacteria colonize wounds within 48 hours after injury and bacteria such as *Staphylococcus aureus, Pseudomonas aeruginosa, *and *Streptococcus* spp may cause infection and this may prolong inflammatory phase of wound healing [3]. Therefore suitable antimicrobial agents can be used either topically or systematically to prevent infection of wounds and speed up wound healing process.

The process of inflammation normally leads to the release of biologically active mediators to attract neutrophils, leucocytes and monocytes, to the wound area and these attack foreign debris and microorganisms through phagocytosis. This then leads to the production of oxygen-free radicals such as hydrogen peroxide, superoxide anion, and hydroxyl anion and excess of these agents causes tissue damage in man or animal if they overwhelm the natural antioxidants of the host such as catalase, superoxide dismutase, and glutathione peroxidase. Therefore, antioxidants prevent the activity of the free radicals and thereby prevent the damage to cells and tissues, providing protection to human and animal subjects, and also enhance healing of infected and noninfected wounds [3, 4].

*Kigelia africana* (Lam.) Beneth. belongs to the family Bignoniaceae. It is known as “Nufutene” in local Asante-Twi in Ghana. It is widespread across Africa including Ghana, Sierra Leone, Gambia, Sudan, and Nigeria and it is found in wet savannah and near river bodies where it occurs in abundance [5]. It is used to treat skin ailments including fungal infections, boils, psoriasis and eczema, leprosy, syphilis, and cancer. The roots, the wood, and leaves have been found to contain kigelinone, vernolic acid, kigelin, iridoids, luteolin, and 6-hydroxyluteolin [6]. The iridoids have antibacterial effect [7]. 

*Strophanthus hispidus *DC. belongs to the family Apocynaceae and it is called “Maatwa” in the local Asante-Twi. It is found all over Africa and savannah forests in Ghana, Senegal, Sudan, Congo DR, Uganda, and Tanzania. It has many medicinal uses such as antidote to the poison of the black-necked cobra, in treatment of syphilis ulcers, bony syphilis, and guinea-worm sores and wounds [1]. The plant contains an amorphous glycoside (pseudo-strophanthin) with heavy oil, two alkaloids (trigonelline and choline), resin, mucilage, and a rhamnose sugar [5]. The aim of the study is to investigate the antimicrobial, antioxidant, and wound healing properties of methanol leaf and stem bark extracts of *K. africana* and methanol leaf and root extracts of *S. hispidus*.

## 2. Materials and Methods

### 2.1. Plant Materials and Chemicals

Stem bark and leaves of *K. africana* and leaves and roots of *S. hispidus* were collected on May, 2011 from Krofrom in the Atwima-Kwanwoma district of Ashanti Region and authenticated by Dr. A. Asase of Ghana Herbarium, Department of Botany, University of Ghana. Voucher specimens of the plants have been deposited at Ghana Herbarium, University of Ghana, Ghana. The various plant parts were dried at room temperature (28–30°C) for two weeks. The dried plant parts were then milled into powdered materials. Unless stated otherwise, all the chemicals were purchased from Sigma (Deisenhofen, Germany).

### 2.2. Preparation of Extracts

Twenty grams of powdered* K. africana* leaves were added 300 mL of 70% methanol and extracted with Ultra-Turrax T 50 (Janke & Kunkel, Labortenik, Germany) under ice-cooling at a speed of 24000 rpm for 3–5 min. The resultant mixture was then filtered using Whatmann filter paper No.10. The rotary evaporator was then used to concentrate the supernatant below 40°C and lyophilized. The procedure was repeated for all the remaining powdered plant materials (*K. africana* stem bark, *S. hispidus* leaves and roots). The yields of the leaf extract (KAL) and stem bark extract (KASB) of *K. africana* and leaf extract (SHL) and root extract (SHR) of *S. hispidus* were 4.3, 12.8, 13.0, and 11.4% w/w (related to the dried material) respectively.

### 2.3. Preliminary Phytochemical Screening

Phytochemical screening was conducted on the methanol leaf and stem bark extracts of *K. africana* and leaves and roots of *S. hispidus* to ascertain the presence of starch, tannins, glycosides (sapogenetic, anthracene, and cyanogenetic), flavonoids, steroids, and alkaloids [8, 9]. The tannins content was determined according to the methods of Glasl [10] and using pyrogallol (Merck, Darmstadt, Germany, purity 99.5%, HPLC) as reference compound.

### 2.4. HPLC Finger-Printing of Extracts

The HPLC finger-printing of the extracts (KAL, KASB, SHL, and SHR) was performed on a Thermo Finnigan HPLC system using Hypersil Gold C_18_, reversed-phase column (150 × 4.6 mm). The concentration of extracts was 10 mg/mL. HPLC optimum conditions: Injection volume: 10 *μ*L, Detection wavelength: 254 nm, Mobile phase: methanol : water/50 : 50 (isocratic condition), Temperature: 22°C, Pump pressure: 28 MPa, Flow rate: 1 mL/min, and running time: 10 min.

### 2.5. Determination of Antimicrobial Activity of Extracts

#### 2.5.1. Antimicrobial Susceptibility Test

The antimicrobial activities of the extracts (KAL, KASB, SHL, and SHR) and reference drugs (chloramphenicol and clotrimazole (Sigma, Deisenhofen, Germany) were determined according to the method described by Agyare and his colleagues [9]. Nutrient agar (Oxoid Limited, United Kingdom) and sabouraud agar (Oxoid Limited, United Kingdom) media were used for both determinations of antibacterial and antifungal activities, respectively. One hundred microliter (10^6^ cfu/mL) of the test organisms (*Escherichia coli *ATCC 25922, *Pseudomonas aeruginosa *ATCC 27853, *Staphylococcus aureus* ATCC 25923, *Bacillus subtilis *NCTC 10073, and clinical fungal agent *Candida albicans*) were used to seed nutrient agar and sabouraud agar plates, respectively. In each of these plates, four (4) equidistant wells with diameter of 8 mm were cut out using sterile cork borer and wells filled with different concentrations of extracts and reference drugs dissolved in dimethyl sulfoxide (DMSO) and allowed to diffuse at room temperature (28–30°C) for 1 h. The zones of growth inhibition were measured after 24 h incubation at 37°C (for the bacteria) and 3 days at 30°C (for the fungus). The activity of DMSO was determined and was found to exhibit no activity against the test organisms. 

#### 2.5.2. Microdilution Method

The MICs of the extracts (KAL, KASB, SHL, and SHR) against the test bacteria were determined using the modified microdilution technique as described by Agyare et al. [9] and Eloff [11]. Test solutions (100 mg/mL) of the extracts were prepared with DMSO and test solution (25–100 *μ*L) was serially diluted to 100 *μ*g/mL and 100 *μ*L (10^6^ cfu/mL) of the test bacteria grown in nutrient broth (Oxoid Limited, United Kingdom) added to each well in the microplates. The covered microplates were incubated at 37°C for 24 h. To indicate growth, 30 *μ*L of 3-(4,5-dimethylthiazol-2-yl)-2,5-diphenyltetrazolium bromide [MTT, thiazolyl blue] dissolved in water was added to the microplate wells and incubated at 37°C for 30 min. Test fungal agent (*C. albicans*) was cultivated in sabouraud dextrose broth (Oxoid Limited, United Kingdom) and then incubated for 3 days at 30°C. The MICs of KAL, KASB, SHL, and SHR extracts against the test fungus were determined according to the guidelines described in the National Committee for Clinical Laboratory Standards [12] for filamentous fungi. The minimum inhibitory concentrations of the extracts against the test organisms were detected as the minimum concentration of extracts that did not exhibit microbial growth after the addition of MTT to the medium and incubation at 37°C for 20 min [9, 13]. The above experiments were repeated three times.

### 2.6. Determination of Free Radical Scavenging Activity

The free radical scavenging activities of the extracts were determined according to method of Chizzola and his colleagues [14] using 1, 1-diphenyl-2-picryl-hydrazyl (DPPH). Solution (0.1 mM) of DPPH in methanol was prepared and 10 *μ*L of this solution were added to 100 *μ*L of methanol extracts together with *α*-tocopherol at different concentrations in 96-well microtitre plates. The plates were shaken for 30 sec and after 30 min the absorbance was measured at 517 nm. The inhibition percentage (%) of radical scavenging was calculated using the following equation. Inhibition (%) = [(*A*_0_ − *A*_1_)/*A*_0_] × 100, where *A*_0_ is the absorbance of the control, *A*_1_ is the absorbance of the sample at 517 nm and Inhibitory Concentration, IC_50_ is the amount (*μ*g/mL) reducing the absorbance by 50%.

### 2.7. Evaluation of Wound Healing Properties (Excision Wound Model)

#### 2.7.1. Experimental Animals

Thirty-five (female Sprague Dawley) rats were housed in stainless steel cages and fed with normal commercial rats diet (GAFCO, Tema, Ghana), given water *ad libitum* and maintained under laboratory conditions (temperature 28–30°C, relative humidity 60–70%, and normal light-dark cycle). A day before the experiment, the rats were brought to the laboratory and habituated to experimenter handling and the apparatus to minimize the effect of stress and novelty. All procedures and techniques used in these studies were in accordance with the National Institute of Health Guidelines for the Care and Use of Laboratory Animals (NIH, Department of Health Services publication No. 83-23, revised 1985). The protocols for the study were approved by the Departmental Ethics Committee.

#### 2.7.2. Excision Wound Model

The animals (female Sprague Dawley rats) weighing 115–120 g were anaesthetized with ketamine at 120 mg/kg body weight subcutaneously prior to the creation of the wounds. The dorsal fur of the animals was shaved to a circular diameter of about 40 mm by means of razor blades and the anticipated area of the wound to be created was outlined on the shaved skin. The areas were cleaned with 70% ethanol before the excision wounds were created according to the modified method of Bhakta et al. [15]. Skin wounds were created along the markings using toothed forceps, surgical blades, and pointed scissors. The entire wounds were left opened and the animals divided into seven (7) groups of five animals each. The first group was topically treated with 1% w/w silver sulphadiazine ointment (Arytons Drugs, Ghana) as reference drug [16]. The second group was treated with aqueous cream (vehicle alone). The third group was left untreated and allowed for normal wound healing to take place. The last four groups were treated with 7.5% w/w extract creams (KAL, KASB, SHL, and SHR), respectively. Wound treatment commenced on the 2nd day after wound creation. The extracts and reference drugs were topically applied to the wounds 24 hourly for 24 days. In the course of treatment, scaled photographs of the wound areas were taken (by means of high resolution Digital Camera) alongside a millimeter scale measurement every 48 h starting from the first day of wound treatment. The wound areas were determined every two days till the 24th day.

### 2.8. Histopathological Studies

Wound tissue specimens from untreated and treated animals were taken during healing process at day 14. The cross sectional full thickness wound scar, of about 6 mm thick sections from each group, was collected at the end of the experiment to evaluate the histopathological alterations [17]. Samples were fixed in 10% buffered formalin for 24 h and dehydrated with a sequence of ethanol-xylene series of solutions, processed and blocked with paraffin at 40–60°C and then sectioned into 5-6 *μ*m thick sections. The sections were stained with hematoxylin and eosin stain, Van Gieson's stain, and toluidine blue stain. Hematoxylin and eosin stained sections and Van Gieson's stained sections were checked for collagen deposition. Toluidine blue stained sections were used to stain mast cells [18].

### 2.9. Statistical Analysis

GraphPad Prism Version 5.0 for Windows (GraphPad Software, San Diego, CA, USA) was used for all statistical analyses. Data are presented as mean ± SEM (*N* = 5) and analyzed by One-way ANOVA followed by Dunnet's multiple comparison's test. *P < 0.05, **P < 0.01, and ***P < 0.001 were considered statistically significant in all analyses. The graphs were plotted using Sigma Plot for Windows Version 11.0 (Systat Software Inc., Germany).

## 3. Results

### 3.1. Preliminary Phytochemical Screening

Both the leaf and stem bark of *K. africana *and *S. hispidus* were found to contain tannins (with varying amounts), steroids, saponins, sapogenetic glycosides, and carbohydrates while the leaves of the two plants contain flavonoids. Alkaloids were present in both leaf and root of *S. hispidus* (Table 1). 

### 3.2. HPLC Finger-Printing of Extracts

**Figure 1:**
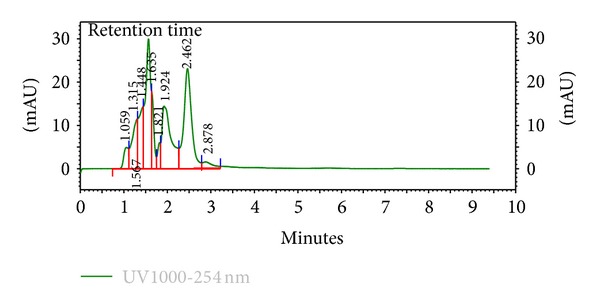
HPLC chromatogram (finger-printing) of methanol leaf extract (KAL) of *K. africana *at *λ* 254 nm.

**Figure 2:**
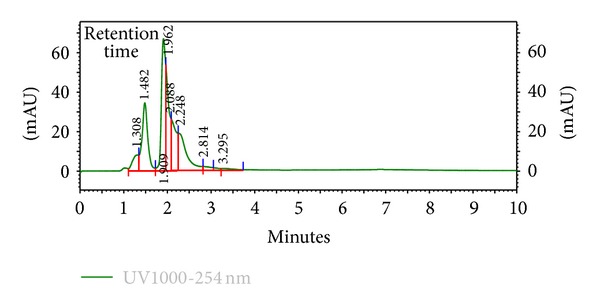
HPLC chromatogram (finger-printing) of methanol stem bark extract (KASB) of *K. africana *at *λ* 254 nm.

**Figure 3:**
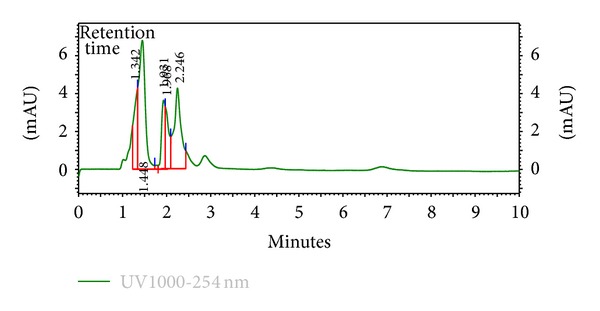
HPLC chromatogram (finger-printing) of methanol leaf extract (SHL) of *S. hispidus *at *λ* 254 nm.

**Figure 4:**
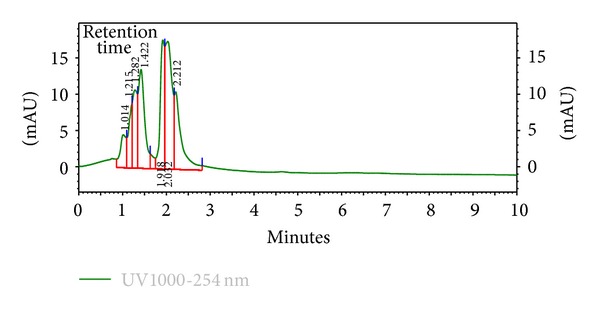
HPLC chromatogram (finger-printing) of methanol root extract (SHR) of *S. hispidus* at *λ* 254 nm.

The HPLC finger-printing of the extracts (KAL, KASB, SHL, and SHR) was determined to identify the major peaks (compounds) in the various extracts for the purposes of identification and quality control (Figures 1, 2, 3, and 4).

### 3.3. Antimicrobial Activity

The methanol extracts (KAL, KASB, SHL, and SHR) were found to be active against the test organisms (*E. coli*, *P. aeruginosa, S. aureus*,* B. subtilis, *and* C. albicans*) with varying mean zones of inhibition and* P. aeruginosa* was found to be less susceptible to the extracts. The minimum inhibitory concentration ranges of *K. africana* extracts (KAL and KASB) against the test organisms were from 2.25 to 7.5 mg/mL and that of *S. hispidus* extracts (SHL and SHR) were 2.5 to 10 mg/mL (Table 2). With respect to the agar diffusion method, all the extracts (KAL, KASB, SHL, and SHR) with concentrations of 20 and 50 mg/mL exhibit higher zones of inhibition against the test organisms than 10 mg/mL concentration (Table 3). 

### 3.4. Antioxidant Activity

**Figure 5:**
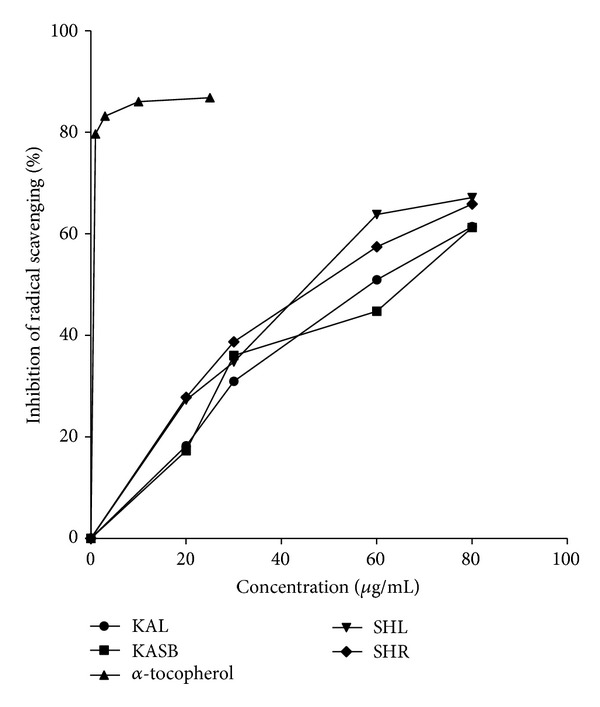
Free radical scavenging activities of methanol leaf extract (KAL) and stem bark extract (KASB) of *K. africana* and leaf extract (SHL), root extract (SHR) of *S. hispidus* and *α*-tocopherol (reference antioxidant) determined by DPPH method.

All the extracts showed some level of antioxidant properties with KASB having lowest IC_50_ and KAL with the lowest free scavenging activity (Table 4 and Figure 5).

### 3.5. Wound Healing Activity (Rate of Wound Closure)

**Figure 6:**
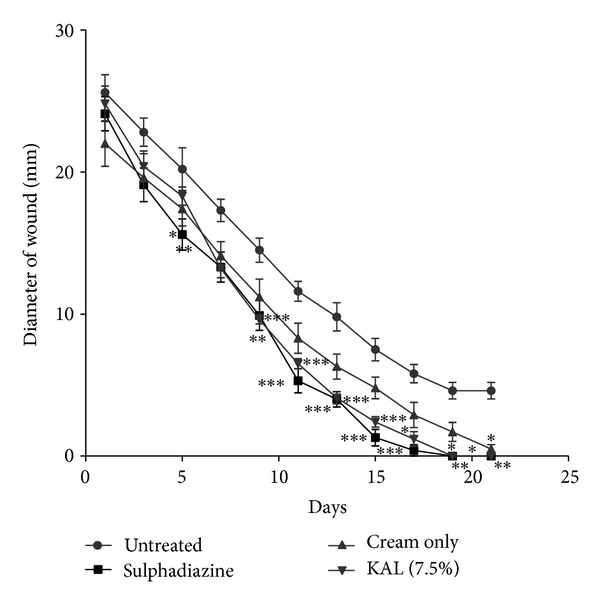
Influence of methanol leaf extract (KAL) cream (7.5%  w/w) of *K. africana *on the rate of closure of wounds. Values are diameter of wounds expressed as mean ± SEM (*N* = 5). *P < 0.05, **P < 0.01, and ***P < 0.001. Control is the untreated wounds. 1% w/w silver sulphadiazine was used as positive control.

**Figure 7:**
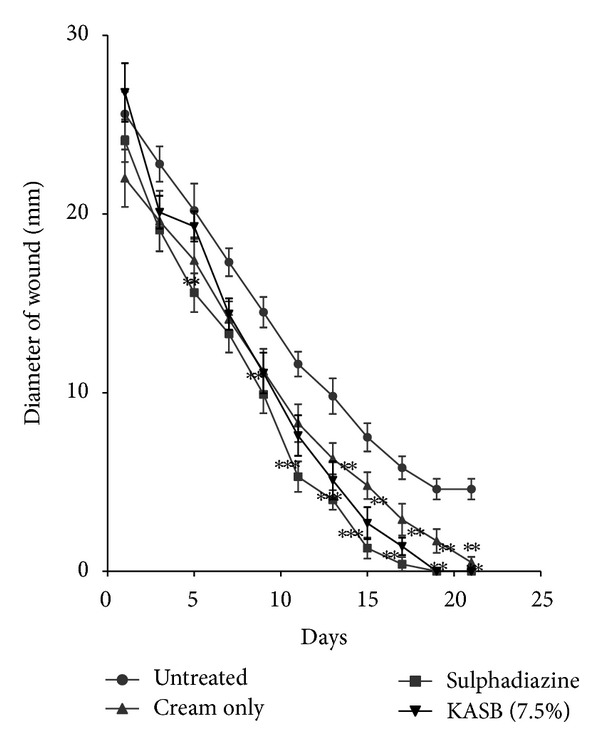
Influence of methanol stem bark extract (KASB) cream (7.5% w/w) of *K. africana *on the rate of closure of wounds. Values are diameter of wounds expressed as mean ± SEM (*N* = 5). *P < 0.05, **P < 0.01, and ***P < 0.001. Control is the untreated wounds. 1% w/w silver sulphadiazine was used as positive control.

**Figure 8:**
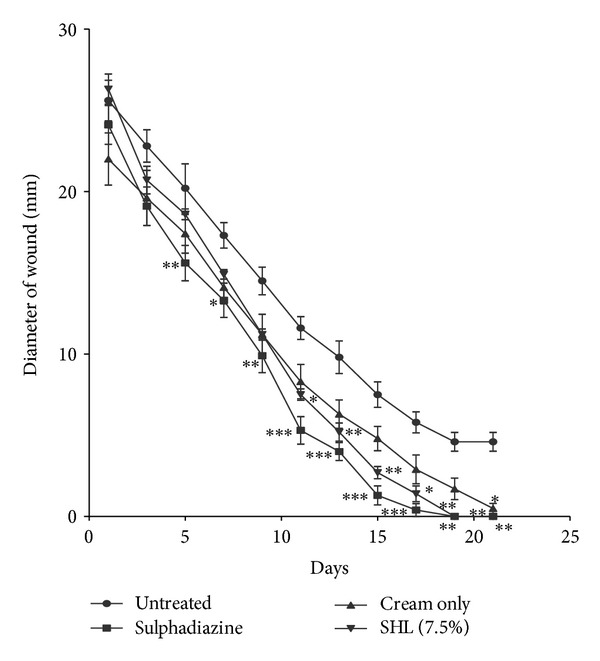
Influence of methanol leaf extract (SHL) cream (7.5%  w/w) of *S. hispidus *on the rate of closure of wounds. Values are diameter of wounds expressed as mean ± SEM (*N* = 5). *P < 0.05, **P < 0.01, and ***P < 0.001. Control is the untreated wounds. 1% w/w silver sulphadiazine was used as positive control.

**Figure 9:**
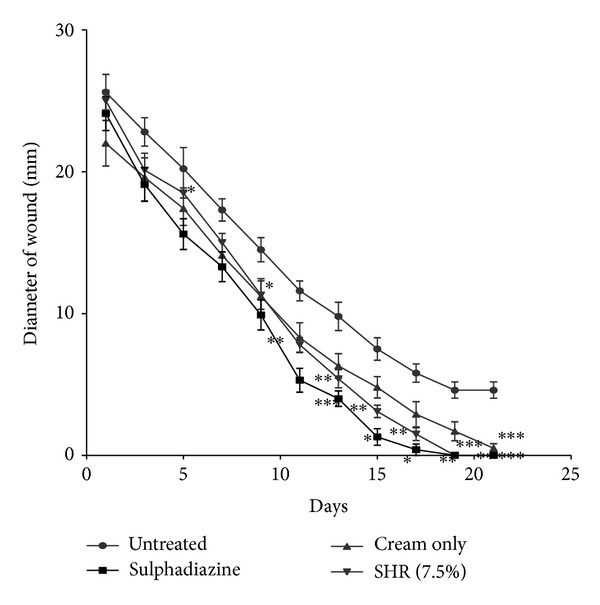
Influence of methanol root extract (SHR) cream (7.5%  w/w) of *S. hispidus *on the rate of closure of wounds. Values are diameter of wounds expressed as mean ± SEM (*N* = 5). *P < 0.05, **P < 0.01, and ***P < 0.001. Control is the untreated wounds; 1% w/w silver sulphadiazine was used as positive control.

All the extracts (KAL, KASB, SHL, and SHR) treated groups exhibited significant activities on the rates of wound closure compared to the untreated and the vehicle alone with KAL and SHL having significant influences (*P* < 0.05 and *P* < 0.01, resp.) on the rate of wound closure from 7th to 15th day after treatment (Figures 6 and 8, Table 5) and KASB and SHR exhibiting significant similar effects on wound healing from 10th to 18th day after treatment (Figures 7 and 9, Table 5). 

### 3.6. Histopathological Studies

**Figure 10:**
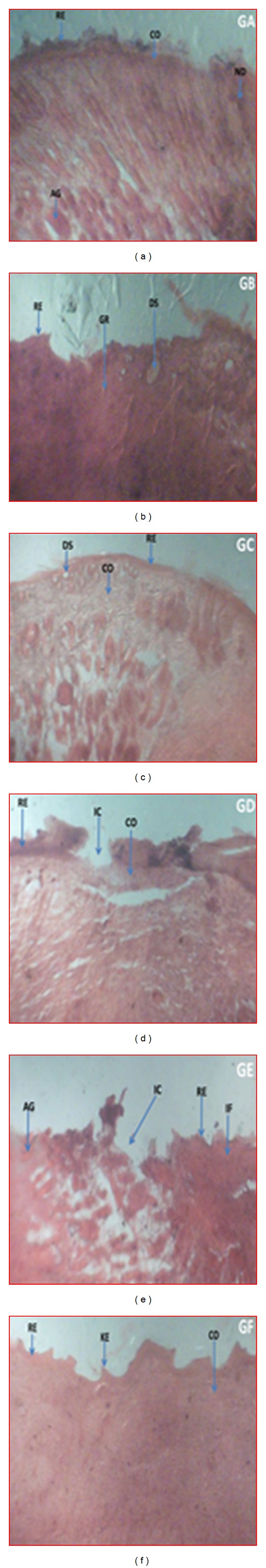
Histopathological examination of wound tissues treated with vehicle, extracts, and the untreated tissues. Representative images stained with hematoxylin and eosin stain, Van Gieson's stain and toluidine blue stain treated daily with 7.5%  w/w creams of methanol leaf extract (KAL) of *K. africana* (a), methanol stem bark extract (KASB) of *K. africana* (b), methanol leaf extract (SHL) cream of *S. hispidus* (c) and methanol root extract (SHR) cream of *S. hispidus *(d) and the untreated wound tissues (e) for 14 days. (a) KAL: profuse angiogenesis, enhanced collagenation, and reepithelialization, evident of marked treatment responses amidst persistent inflammation. (b) KASB: appreciable angiogenesis and granulation tissue formation with evidence of apoptosis following tissue necrosis with less intense collagenation and reepithelialization. (c) SHL: With appreciable intense collagenation and reepithelialization. (d) SHR: With appreciable granulation tissue formation manifested as tissue refilling. Collagenation and re-epithelialization were also in earnest compared to the untreated wound tissue. (e) Untreated wound tissues with persistent inflammation with incomplete wound area; evidence of poor granulation tissue formation, collagenation and reepithelialization but profuse angiogenesis. (f) Treated wound tissues with 1% w/w silver sulphadiazine showed rapid granulation tissue formation, collagenation, evident of enhanced wound healing and uneven keratinous wound surface evident reepithelialization. Legend: AG: angiogenesis, CO: collagenation, DS: dead space following necrosis, GR: granulation tissue following apoptosis, IC: incomplete wound area, IF: inflamed tissue, KE: keratinous epithelium, ND: necrotic debris of persistent inflammation. RE: reepithelialisation.

Histological studies revealed profuse proliferation of fibroblasts with varying degree of fibrosis. The specimens showed 70 to 80% dense and thickened fibrosis for the wounds treated with the extracts while the 1% w/w silver sulphadiazine ointment (positive control) showed 60 to 70% fibrosis. Fibroblast cells and collagen fibers were prominently present in the reference and extracts treated groups as compared to untreated control. There were profuse angiogenesis, enhanced collagenation, and reepithelialization, evidence of marked treatment responses amidst persistent inflammation with wound tissues treated with the extracts compared to the untreated wound tissues (Figure 10).

## 4. Discussion

The present investigation describes some of the biological activities including antimicrobial and wound healing properties of the leaves, stem bark, and roots extracts from the tropical plants *K. africana* and *S. hispidus*. Plant products are potential wound healing agents, and largely preferred because of their widespread availability, less or no side effects, and effectiveness as crude preparations [2]. The phytochemical screening of the dried leaves and root of *S. hispidus* revealed the presence of tannins, alkaloids, saponins, steroids, carbohydrates and sapogenetic glycosides, and flavonoids in the leaves. Alkaloids were absent in the dried leaves and stem bark of *K. africana* and saponins, steroids, carbohydrates, and sapogenetic glycosides were present in both plant materials. Flavonoids were also found in the leaves of *K. africana* (Table 1). The HPLC finger-printing of the extracts (KAL, KASB, SHL, and SHR) was also developed for identification and quality control purposes (Figures 1, 2, 3, and 4).

The phytochemical constituents of a plant often determine the physiological action on the human body. Antioxidants are agents that protect cells against damage caused by molecules known as free radicals. The antioxidant activities of extracts are mainly due to the presence of phenolic compounds such as flavonoids, phenolic acids, tannins, and phenolic diterpenes [19]. Hence, the constituents of the extracts, such as tannins and flavonoids, play a major role in the wound healing by preventing and protecting oxidative damage from free radicals [20, 21].

The antimicrobial activity of the methanol extracts of *K. africana *leaves and stem bark and *S. hispidus *leaves and roots was determined against four bacteria, two Gram-positive bacteria (*S. aureus* and *B. subtilis*), two Gram-negative bacteria (*E. coli* and *P. aeruginosa*), and one fungus (*C. albicans*), using the cup plate agar diffusion method. The methanol extracts of the two plants were active against all the tested organisms (Table 2). The minimum inhibitory concentration (MIC) was determined as the lowest concentration of crude extract at which no microbial growth and MICs of KAL against *S. aureus, B. subtilis, E. coli, P. aeruginosa,* and *C. albicans* were 5, 2.5, 5.5, 7.5, and 2.5 mg/mL, respectively and that of KASB were 5, 5.5, 5.25, 7.5, and 2.25 mg/mL, respectively. SHL and SHR extracts also exhibited similar antimicrobial activity and their MICs were in the same range as the *K. africana* extracts (Tables 2 and 3). The antibacterial and antifungal activities of the extracts (KAL, KASB, SHL, and SHR) were similar to activity exhibited by leaf extracts of *Kigelia pinnata* (Jacq.) DC. as reported by Binutu et al. [22]. The antimicrobial action of the extracts may be attributed to astringent nature of the phenolic constituents including tannins and other polyphenols present in the extracts [23]. 

The inhabitation of pathogenic bacteria such as *Staphylococcus, Streptococcus*, and *Pseudomonas* in wounds normally may lead to infection of wounds which may result in the formation of chronic wounds [24]. From this study, it was realized that the *K. africana* and *S. hispidus* extracts exhibited strong and broad spectrum antimicrobial activity against these pathogens. Since most of the MICs of the extracts against the test organisms were below 8 mg/mL, it could be inferred that the extracts exhibited potent antimicrobial activity according to Fabry and his colleagues [25]. The topical application of antimicrobial agents or extracts is an efficient therapy method of destroying microbial populations because of the availability of the active agents at the wound site which leads to enhanced wound healing activity [20, 21]. 

The IC_50_ values of the extracts (KAL, KASB, SHL, and SHR) were 1.5, 56.9, 13.7, 49.8, and 45.1 *μ*g/mL, respectively (Table 4). These results suggest that these extracts possess antioxidant properties with exception of extracts of* S. hispidus* and this could facilitate healing of wounds [3]. This may indicate that the traditional uses of *S. hispidus* as wound healing agent may not be necessarily due to its antioxidant activity but rather be based on other biological effects. The IC_50_ values of leaves and stem bark of *K. africana *were similar to findings of Gathirwa and his colleagues [26].

The extracts (KAL, KASB, SHL, and SHR) had significant influence on the rate of wound closure based on the different days of treatment of the wounds with the extracts compared with the untreated. SHL extract exhibited significant enhanced effect on the wound healing process at day 11 (*P* < 0.05) with percentage wound closure of 90.13 (Figure 8) compared with the untreated wounds. The influence of SHL on the wounds was similar to that of SHR extract with significant increased rate of wound contraction on day 11 (*P* < 0.05) and wound closure of 91.72% (Figure 9). The influence of KASB extract (Figure 7) was similar to SHL and SHR extracts. However, KAL extract significantly improved wound contraction from day 7 (*P* < 0.05) with wound closure of 85.1% (Figure 6) to the 17th day. Histopathological examination of the wound tissues revealed profuse angiogenesis, enhanced collagenation, and reepithelialization compared to the untreated wounds (Figure 10). These biological activities of the extracts may be due to the enhanced proliferation of fibroblasts and keratinocytes [27] and successful reduction of pathogenic bacteria by the extracts [28] and these may be attributed to the phytochemical constituents of the extracts. 

The effects observed in cream only treated and the untreated wounds were not statistically significant compared to the extracts or silver sulphadiazine (reference) treated wounds. This may also indicate that the components of the aqueous cream did not interfere with the activity of the extracts and hence the enhanced effects of the extracts may be solely due to the bioactive principles present in these extracts. The wound healing effects of the extracts may be due the various phytochemical constituents present in them. Tannins such as proanthocyanidins and other tannins including tannic acid, geraniin, and furosin are known to facilitate wound healing [27, 29–31]. The extracts were found to contain flavonoids and saponins and these secondary metabolites have been found to enhance wound healing [31, 32] and hence the enhanced wound healing effects of the extracts may be attributed to their phytochemical constituents.

The above findings may support the claims that wounds treated with plant extracts heal faster and better than untreated wounds [22, 33] and the use of these plants for the treatment of microbial infections. However, isolation and characterization of bioactive compound(s) responsible for these pharmacological properties need to be performed.

## 5. Conclusion

The methanol leaf and stem bark of *K. africana *and methanol leaf and root extracts of *S. hispidus *exhibited antioxidant, antimicrobial activities with MIC ranges of 2.25 to 7.5 mg/mL and 2.5 to 10 mg/mL, respectively, against the test organisms, enhanced wound healing properties and these pharmacological properties may justify the medicinal uses of these plants for treatment of microbial infections and wounds. Bioactivity guided fractionation and isolation of the bioactive compounds responsible for the biological activities would be carried out.

## Figures and Tables

**Table 1:** Preliminary phytochemical screening of dried leaves and stem barks of *K. africana* (KA) and *S. hispidus* (SH).

Plant material/part	Secondary metabolites						
	Alkaloids	Saponins	Flavonoids	Steroids	Carbohydrates	Sapogenetic glycosides	Tannins (% w/w)
KA leaf	−	+	+	+	+	+	0.95
KA stem bark	−	+	−	+	+	+	1.58
SH leaf	+	+	+	+	+	+	1.32
SH root	+	+	−	+	+	+	1.22

+: presence of secondary metabolite; −: absence of secondary metabolites.

**Table 2:** Minimum inhibitory concentrations (MICs) of methanol leaf extract (KAL) and stem bark extract (KASB) of *K. africana* and leaf extract (SHL) and root extract (SHR) of *S. hispidus* determined by microdilution method. The experiments were repeated three times. Reference antimicrobial agents: CPC: chloramphenicol, CTZ: clotrimazole, nd: not determined.

Extract/MIC (mg/mL)	*S. aureus* ATCC 25923	*B. subtilis* NCTC 10073	*E. coli* ATCC 25922	*P. aeruginosa* ATCC 27853	*C. albicans *
KAL	5.0	2.5	5.5	7.5	2.5
KASB	5.0	5.5	5.25	7.5	2.25
SHL	3.25	7.5	5.5	7.5	2.5
SHR	5.5	2.5	5.5	10.0	5.5
CPC	0.025	0.020	0.025	0.055	nd
CTZ	nd	nd	nd	nd	0.025

**Table 3:** Antimicrobial activity of methanol leaf extract (KAL) and stem bark extract (KASB) of *K. africana* and leaf extract (SHL) and root extract (SHR) of *S. hispidus* by agar diffusion method. Mean zones of growth inhibition (plus diameter of well) are mean (mm) of 3 independent experiments, mean ± SD, *n* = 3 replicates, diameter of well/cup = 8 mm, reference antimicrobial agents: CPC: chloramphenicol (1 mg/mL), CTZ: clotrimazole (1 mg/mL), nd: not determined.

Mean zones of growth inhibition (mm)					
Extract (mg/mL)	Test organisms				
	*S. aureus* ATCC 25923	*B. subtilis* NCTC 10073	*E. coli* ATCC 25922	*P. aeruginosa* ATCC 27853	*C. albicans *
KAL					
10	12.45 ± 0.50	12.50 ± 0.40	12.00 ± 0.50	0.0	15.50 ± 0.50
20	14.50 ± 0.50	13.53 ± 0.50	15.50 ± 0.25	12.50 ± 0.70	18.50 ± 0.55
50	16.35 ± 0.45	16.50 ± 0.35	19.65 ± 0.55	15.50 ± 0.70	23.35 ± 0.45
KASB					
10	0.0	11.50 ± 0.25	12.50 ± 0.50	0.0	12.55 ± 0.55
20	13.00 ± 0.50	13.50 ± 0.45	16.55 ± 0.25	10.50 ± 0.50	15.50 ± 0.50
50	15.00 ± 0.55	15.00 ± 0.50	19.65 ± 0.25	14.50 ± 0.25	18.5 ± 0.25
SHL					
10	11.00 ± 0.50	0.0	11.00 ± 0.50	0.0	12.50 ± 0.25
20	13.50 ± 0.55	12.50 ± 0.50	13.25 ± 0.50	14.50 ± 0.25	14.25 ± 0.25
50	16.55 ± 0.25	14.50 ± 0.25	17.50 ± 0.25	16.55 ± 0.50	18.25 ± 0.50
SHR					
10	12.5 ± 0.50	12.55 ± 0.50	11.25 ± 0.50	0.0	12.50 ± 0.20
20	14.50 ± 0.50	15.50 ± 0.50	15.55 ± 0.50	13.00 ± 0.50	14.50 ± 0.50
50	18.25 ± 0.25	17.50 ± 0.25	17.00 ± 0.25	15.50 ± 0.25	16.50 ± 0.25
CPC	25.5 ± 0.25	31.50 ± 0.25	30.55 ± 0.25	17.50 ± 0.25	nd
CTZ	nd	nd	nd	nd	25.50 ± 0.50

**Table 4:** Free radical scavenging activities of methanol leaf extract (KAL) and stem bark extract (KASB) of *K. africana* and leaf extract (SHL), root extract (SHR) of *S. hispidus* and *α*-tocopherol determined by DPPH method.

Extracts	IC_50_ (*μ*g/mL)
KAL	56.9
KASB	13.7
SHL	49.8
SHR	45.1
*α*-tocopherol	1.5

**Table 5:** Summary of wound closures for selected time points. Values are mean wound area (mm^2^) ± SEM, for untreated wounds and wounds treated with 1% w/w silver sulphadiazine, 7.5% w/w* K. africana *extract cream (KAL), and stem bark extract (KASB) of *K. africana* and leaf extract (SHL), root extract (SHR) of *S. hispidus* and cream only. *N* = 6 rats per group. Data analysed by one-way ANOVA followed by Dunnet's multiple comparison's test.

Day	Untreated wounds	Silver sulphadiazine	Cream	KAL	KASB	SHL	SHR
1	519.6 ± 113.0	460.7 ± 100.8*	388.2 ± 119.7*	488.0 ± 108.1	572.6 ± 162.2	538.0 ± 89.4	492.5 ± 62.9
3	400.6 ± 73.0	290.9 ± 77.6**	310.7 ± 111.5	330.5 ± 76.9	319.9 ± 66.2	338.8 ± 60.7	319.7 ± 63.0
5	375.5 ± 117.9	194.9 ± 58.6*	242.3 ± 74.1	264.3 ± 41.5	294.8 ± 58.7	272.1 ± 21.5	269.2 ± 23.0*
7	237.0 ± 45.9	142.4 ± 52.2**	159.32 ± 51.24	138.2 ± 30.9*	165.3 ± 44.9	174.6 ± 15.2	178.0 ± 34.4
9	167.4 ± 42.9	80.5 ± 39.5***	103.4 ± 48.0	72.7 ± 9.9**	100.8 ± 42.8	98.9 ± 13.0	103.5 ± 41.5*
11	112.7 ± 28.6	24.3 ± 18.7***	57.6 ± 29.4	33.6 ± 8.1***	49.4 ± 29.3*	44.6 ± 9.3*	48.6 ± 14.5
13	73.00 ± 26.54	13.5 ± 8.7***	33.6 ± 18.9	13.8 ± 6.5***	24.0 ± 18.8**	22.3 ± 10.0***	24.2 ± 11.9**
15	46.1 ± 20.1	2.4 ± 2.9***	19.9 ± 12.1	5.0 ± 3.3***	8.2 ± 8.1**	6.2 ± 3.4**	8.1 ± 5.0
17	27.7 ± 13.2	0.6 ± 1.4***	9.1 ± 8.3	2.0 ± 2.1**	2.3 ± 2.8*	2.3 ± 2.8**	2.4 ± 2.0**
19	17.7 ± 9.8	0.0**	3.7 ± 4.4	0.0**	0.0**	0.0**	0.0**
21	17.7 ± 9.8	0.0**	0.5 ± 0.8*	0.0**	0.0**	0.0**	0.0**

*P < 0.05, **P < 0.01, ***P < 0.001 were considered statistically significant compared to the untreated wounds.
